# Metabolic syndrome in rural Peruvian adults living at high altitudes using different cookstoves

**DOI:** 10.1371/journal.pone.0263415

**Published:** 2022-02-08

**Authors:** Giuliana Sanchez-Samaniego, Daniel Mäusezahl, Cesar Carcamo, Nicole Probst-Hensch, Héctor Verastegui, Stella Maria Hartinger

**Affiliations:** 1 Department of Epidemiology and Public Health, Swiss Tropical and Public Health Institute, Swiss TPH, Basel, Switzerland; 2 University of Basel, Basel, Switzerland; 3 School of Public Health and Administration, Universidad Peruana Cayetano Heredia, UPCH, Lima, Peru; 4 Faculty of Science, University of Geneva, Geneva, Switzerland; Tongji Med College, HUST, CHINA

## Abstract

This study determined the prevalence of metabolic syndrome (MetS) in open fire stoves and improved cookstoves users (ICS) in the rural Peruvian Andes. Participants answered a socioeconomic questionnaire, one 24-hour food recall and underwent a physical examination. We analysed data from 385 participants, 190 (112 women and 78 men) were ICS users and 195 (123 women and 72 men) were open fire stove users. The prevalence of MetS was 21.3, 26.4% in women and 13.3% in men. We found no statistically significant association between the type of cookstove and MetS. Body mass index and altitude were important determinants of MetS. Research on cardiometabolic diseases and open fire stove use contributes to understanding the effect of household air pollution on health in high altitude populations.

## Introduction

Non-communicable diseases (NCDs) are the leading cause of death globally and were responsible for 39.5 million deaths worldwide in 2016 [1]. In the last decade, NCDs’ contribution to adult mortality has increased in Peru, particularly due to cardiovascular diseases (CVDs) [2]. The epidemiological transition in Peru has led to the coexistence of communicable and non-communicable diseases, which accounts for differential death rates in different areas of the country [2–4]. Peru’s diverse geography and socioeconomic inequalities add to the differences in disease burden within the country, challenging the disease surveillance and prevention strategies at the sub-national and primary care levels [2, 5]. The lack of resources, evidence-based protocols, access to timely medication and diagnosis are just a few of the bottlenecks that the Peruvian health system faces [6]. Hence, research on local risk patterns for CVDs in remote areas of the country can contribute to national health planning and to the understanding of this epidemiological transition [7].

Metabolic syndrome (Mets) is associated with an increased risk of CVDs and type-2 diabetes mellitus five to ten years post diagnosis [8]. The early identification of individuals with MetS and early prevention strategies can reduce the long-term increase of the risk of CVDs. MetS is defined as the cluster of three or more metabolic abnormalities that includes elevated waist circumference, elevated blood pressure, elevated levels of glucose and triglycerides and low levels of high-density lipoprotein (HDL) cholesterol [8]. Similar to CVDs, MetS is considered a multifactorial health problem that depends on genetic [9–12], metabolic [13, 14], behavioural [15–17], socio-economic [18], and environmental factors [19, 20]. In Peru, MetS’ prevalence varies between 15–55%, depending on sex, region, ethnicity, urbanisation and altitude [21–24].

Exposure to ambient and indoor air pollution and tobacco smoke increases the risk of developing CVDs [19, 20], as well as the risk of MetS [25]. In rural Peru, over 80% of households use biomass for cooking, heating and lighting, thus contributing to high levels of household air pollution (HAP) [26]. Evidence from randomised trials suggests that HAP reductions through the installation of ventilated improved (biomass fuel) cookstoves (ICS) or cleaner fuel stoves using ethanol or liquid petroleum gas (LPG), are associated with lower cardiovascular health risks in women [27–30]. Through national programmes, Non-Governmental organisations and private organisations, Peruvian rural households can now access ICS and cleaner fuels for cooking. However, literature linking CVDs’ burden to ICS interventions is still scarce worldwide, especially among men [27–31].

Our study aims at determining the prevalence of MetS in adults using open fire stoves and ICS installed 11–14 months before this study was conducted in high altitude communities in the rural Peruvian Andes.

## Methods

### Study setting

Our study was conducted in the provinces of San Marcos and Cajabamba in the Cajamarca region of northern Peru. Both provinces are located in the rural Andes between 1900 and 3900 metres above sea level (masl) with a population of more than 134,000 inhabitants [32]. The majority of households are made of adobe walls and have earthen floors. Local trade is the main source of income. The most important agricultural products are potatoes, beans, manioc, rice and wheat, while families typically raise chickens, pigs, ducks, guinea pigs, sheep and goats for self-consumption. These animals are kept in dens but sometimes they can be inside the kitchen or other common areas. Cattle are used for agricultural activity and milk production. Community families can also receive aid from government supplementary feeding, cash transfer and other social programmes.

### Study design

We conducted an exploratory cross-sectional study with adults using open fire stoves and ICS. These adults included mothers and fathers of children who were previously participating in a community-randomised controlled trial focusing on child health outcomes (c-RCT, referred as “parent study”) [33]. The parent study implemented an integrated home-based intervention package (IHIP) which included the installation of a certified ICS model in 80 community clusters. They measured 24-hour kitchen exposure of PM_2.5_ in a subsample of 38 households. The parent study found that the ICS significantly reduced HAP, but not to levels meeting the World Health Organization’s thresholds [33, 34] (S1 Appendix).

All parents of the 317 children participating in the parent study were eligible to participate in this exploratory cross-sectional study. Pregnant women were excluded given pregnancy-related changes in blood pressure or glucose levels. Using an estimated proportion of 50% of MetS in the whole population, we calculated a sample size of 384 adults with a margin error of 5% and a 95% confidence level.

### Enrolment and study participants

Participants were recruited between January and May 2017, which was 11–14 months after the parent study intervention was implemented. Fieldworkers visited the households of the parent study, and invited the eligible adults to participate. Participants were asked to fast overnight prior to the second visit. Upon agreement, an appointment was scheduled. We organised all visits between 5:30–11:00 am at a central location in the community (i.e. schools, community hall) and accordingly to participants’ availability. Occasionally data collection occurred also at a participant’s household. Due to time constraints, we could only visit each community cluster once.

### Data collection

We collected physical measurements (anthropometry, waist circumference and blood pressure), biomarkers for HDL cholesterol, triglycerides and glucose levels through a physical examination, and nutritional data through a 24-hour food recall questionnaire. All field workers were extensively trained in obtaining standardised measurements and administering questionnaires. In addition, we used socio-demographic data and HAP measurements from the parent study.

### Physical examination

Participants underwent a physical examination that included anthropometric, blood pressure, and capillary glucose and lipids measurements. We used standardised techniques for anthropometric examinations [35]. Individuals were weighed using a floor scale. Height was measured using a portable stadiometer. Waist circumference was measured using a tape measure. Blood pressure (BP) was measured with the participant seated and in repose. Participants with high blood pressure (≥130 mm Hg systolic or ≥85 mm Hg diastolic) in their first measurement received a second measurement. Both values were averaged for the MetS diagnosis. We used a calibrated automatic blood pressure meter (OMRON HEM-712C).

We collected biomarkers through fasting capillary blood samples for the analysis of HDL cholesterol, triglycerides and glucose levels. Capillary blood samples were analysed using a point of care testing device (CardioChek PA) validated for reliably measuring lipid and glucose levels [36]. For glucose levels, we applied a conversion factor of 1.11 to transform whole blood glucose values obtained from the device to plasma glucose values [37]. Internal quality control testing was performed as recommended by the manufacturer.

### Dietary recall

Participants answered a 24-hour food recall questionnaire. They were asked to recall all foods and beverages that they consumed the previous day, from the moment they woke up until they went to bed. The questionnaire was adapted from the guideline “Documenting Traditional Food Systems of the Centre for Indigenous Peoples’ Nutrition and Environment” (CINE-McGill University) [38]. We categorised foods in six groups (carbohydrates (cereals, grains and tubers), vegetables, fruits, vegetable protein (legumes), animal protein, dairy products and fats). We calculated the Diet Diversity Score (DDS), defined as the number of food groups consumed by each participant [39]. The DDS ranges from 0 to 7.

### Data abstracted from the parent study

Sociodemographic data of the parent study, including households’ characteristics, education and main economic activity, were used to calculate the Peruvian index of Unsatisfied Basic Needs (NBI, Spanish abbreviation) according to the Peruvian National Institute of Statistics and Informatics [40]. Information on the type of stove used for cooking and heating was also obtained from the parent study.

### Outcome

We used Joint Interim Statement diagnostic criteria to define MetS [8]. Participants were diagnosed with MetS if they presented at least three of the following five risk factors: elevated triglycerides (≥150 mg/dL), reduced HDL cholesterol (≤40 mg/dL for men and ≤50 mg/dL for women), elevated BP (systolic BP ≥130 mm Hg and/or diastolic BP ≥85 mm Hg), elevated fasting plasma glucose (≥100 mg/dL), and elevated waist circumference population specific for Ethnic Central and South Americans (90cm≥ for men and 80cm≥ for women).

### Statistical analysis

Data were entered using the Census and Survey Processing System (CS Pro 6.3) and analysis was performed using STATA 15 Statistical software (STATA CORP, College Station, Texas, USA). Descriptive statistics are presented as mean ± standard deviations for normally distributed data, medians [interquartile range] for non-normally distributed data, and number (percentages) for categorical variables. Pearson Chi-square statistics and Fisher’s exact test were used to assess associations between categorical variables, and Student’s t-test and Wilcoxon rank sum test were carried out for statistical comparisons of continuous data between ICS users and open fire stove users. The significance level was set at p ≤0.05. Subjects with two of five risk factors present and with high blood pressure, constituting the defining criteria to diagnose MetS, were only included in the analysis if two blood pressure measurements could be obtained.

We used a mixed effects Poisson regression with robust variance for the univariable and multivariable analysis to estimate the prevalence ratio (PR) of MetS and its five components [41]. We explored the interaction between the type of cookstove and sex, as women are usually responsible for cooking and spend more time in the kitchen environment compared to men. The interaction term was not significant in the models tested; thus, we did not stratify the analysis by sex and dropped the interaction term in the final multivariable model.

We selected the participants’ household and place of the physical examination as random effects. Fixed effects included: type of cookstove (open fire stove vs ICS), sex, age, agricultural work as main economic activity, education level, altitude of residence, NBI, body mass index (BMI) and DDS in the model. The variable ‘agricultural work’ was used as a proxy for the most demanding physical activity. Smoking status was not included in the analysis as the descriptive results showed that tobacco smoke exposure is low in this population. The multivariable analysis included the exposure variable (type of cookstove) and additional covariates from the univariable analysis with p <0.20.

### Ethics

The study was approved by the Universidad Peruana Cayetano Heredia (UPCH) Ethical Review Board (N° 192-08-16). All participants signed a written informed consent form. Participants received their results after the physical examination. If their results indicated abnormal glucose, lipids and/or blood pressure, they were referred to the local health establishment. This study used data from the parent study, which received ethical clearance earlier (N°R74-15-16 and trial registration number ISRCTN26548981).

## Results

**Fig 1:**
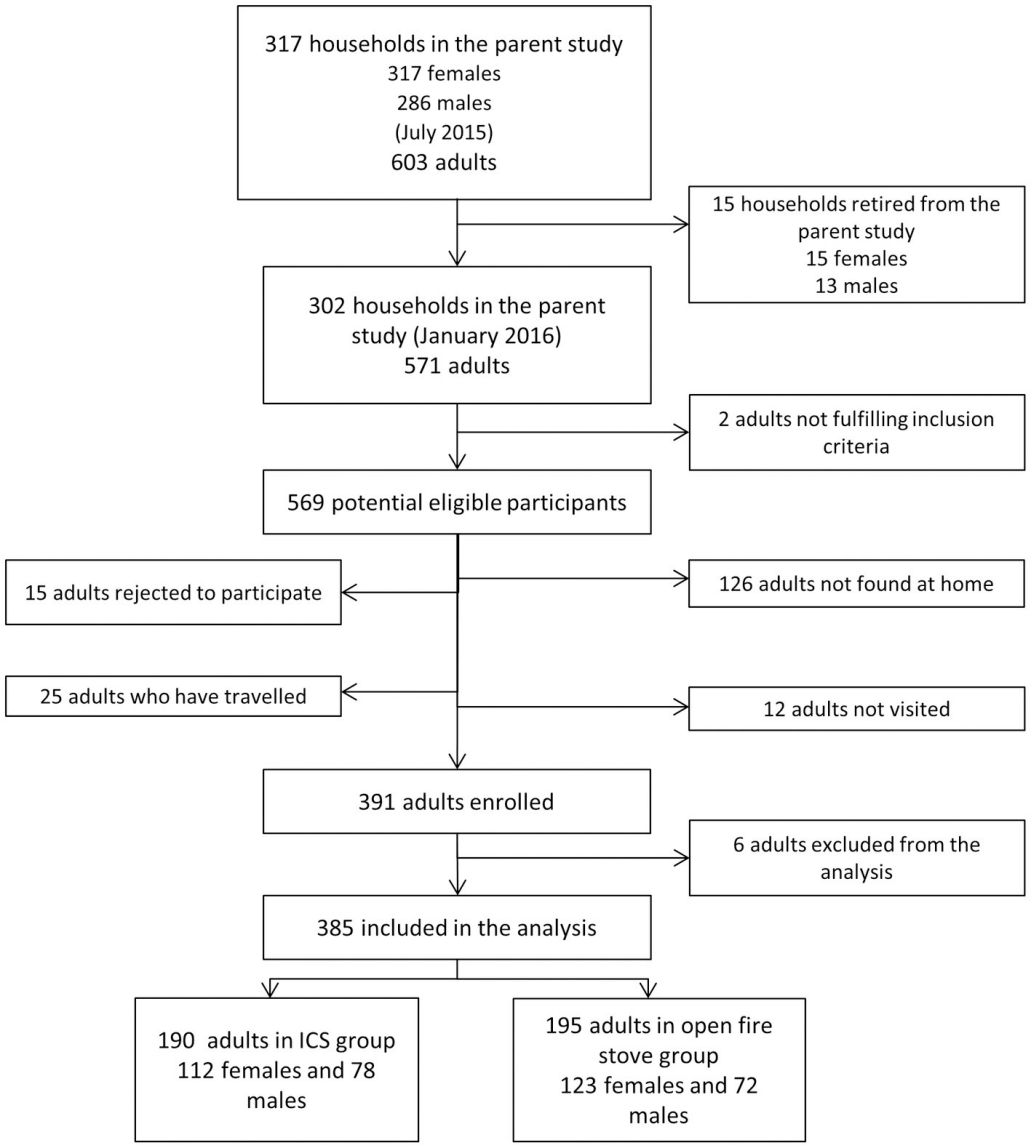
Flowchart of study participants of the provinces of San Marcos and Cajabamba, Cajamarca-Peru. ICS: Improved cookstoves.

The study enrolled 391 adults, 238 (60.1%) women and 153 (39.1%) men, which corresponds to 68% of all eligible participants in the parent study. Of those potential eligible participants, six families could not be invited to participate because heavy rains limited the access to one community; 25 participants were absent travelling; 126 adults were not found at their household (family members had already left for work) at the time of the visit and 15 men rejected participation. Furthermore, of 391 adults, 6 subjects were excluded from the analysis due to an inconclusive diagnosis of MetS. We included 385 adults in the analysis, 190 (49.3%) belonged to the ICS group and 195 (50.7%) to the open fire stove group (Fig 1). The ICS group consisted of 112 women (59.0%) and 78 men (41.0%) and the open fire stove group of 123 (63.1%) women and 72 men (36.9%).

### Population description

Table 1 presents the characteristics of participants of the parent study at baseline and during the physical examination conducted for the purpose of this study. Mean age was similar between ICS and open fire stove groups in both, men (33 ± 7.6 years *and* 34 ± 6.7 years, respectively) and women (30 ± 7.6 year *and* 28 years ± 7.0, respectively). The proportion of women and men with secondary education level or higher and with agriculture as their main activity did not significantly differ between the ICS and open fire stove groups. The physical examinations for the present study were conducted at the participant’s homes in 58% of the ICS users and in 55% of the open fire stove users. Furthermore, 50% of participants in both groups lived at ≥2500 masl. A higher proportion of women in the open fire stove group were obese (17.1%) and overweight (44.7%) compared to women of the ICS group (12.5% and 28.6%, respectively). The proportion of men in the BMI categories did not significantly differ between groups. Regarding the components of MetS, we did not observe a significant difference between these measurements in women and men of both groups.

**Table 1:** Sociodemographic and household characteristics of adult participants of the provinces of San Marcos and Cajabamba, Cajamarca-Peru.

	ICS users	Open fire stove users	p-value
**N (%)**	190 (49.3)	195 (50.7)	
Women	112 (59.0)	123(63.1)	0.406
Men	78 (41.0)	72 (36.9)	
**Participant characteristics**			
Age (years)			
Women	30 ± 7.6	28 ± 7.0	0.176
Men	33 ± 7.6	34 ± 6.7	0.775
Education (secondary level or higher completed) (%)			
Women	15 (13.4)	20 (16.3)	0.537
Men	22 (29.0)	20 (27.8)	0.875
Economic activity (agriculture as main economic activity) (%)			
Women	30 (26.8)	43 (35.0)	0.176
Men	29 (37.9)	30 (41.7)	0.574
Physical examination performed at the participant’s household (%)	110 (58.0)	107 (55.0)	0.550
Altitude of residence ≥2500 masl (%)	95 (50.0)	98 (50.3)	0.960
Body mass index (BMI) (%)			
Women			0.013
Underweight	3 (2.7)	2 (1.6)	
Normal	63 (56.3)	45 (36.6)	
Overweight	32 (28.6)	55 (44.7)	
Obese	14 (12.5)	21 (17.1)	
Men			0.927
Underweight	1 (1.3)	1 (1.4)	
Normal	47 (60.3)	43 (59.7)	
Overweight	25 (32.0)	25 (34.7)	
Obese	5 (6.4)	3 (4.2)	
Glucose (mg/dL)			
Women	88.3 [14.4]	88.8 [17.8]	0.871
Men	86.6 [18.9]	83.2 [11.1]	0.150
HDL cholesterol (mg/dL)			
Women	36.0 [10.0]	35.0 [9.0]	0.694
Men	36.0 [10.0]	35.5 [10.0]	0.400
Triglycerides (mg/dL)			
Women	86.0 [65.0]	91 [61.0]	0.785
Men	84.5 [59.0]	93.5 [68.5]	0.395
Waist circumference (centimetres)			
Women	84.1 [14.2]	86 [12.8]	0.140
Men	84.1 ± 7.0	82.7 ± 7.1	0.222
Systolic blood pressure (mmHg)			
Women	106.4 ± 9.8	106.8 ± 10.8	0.718
Men	115.0 [15.0]	115.0 [11.0]	0.362
Diastolic blood pressure (mmHg)			
Women	70.3 ± 9.0	70.2 ± 7.2	0.954
Men	71.0 [11.0]	73.5 [9.0]	0.078
**Household characteristics**			
Total of households (%)	114 (47.3)	127 (52.7)	
Electricity (%)	81 (71.1)	105 (82.7)	0.032
Adobe walls (%)	105 (92.1)	122 (96.1)	0.190
Roof tiles (%)	96 (84.2)	117 (92.1)	0.056
Earthen floor (%)	99 (86.8)	119 (93.7)	0.070
Latrine (%)	69 (60.5)	78 (61.4)	0.887
Piped water to courtyard (%)	112 (98.2)	126 (99.2)	0.499
Households engaging in community groups or support programmes (%)	78 (68.4)	91 (71.7)	0.584
Unsatisfied basic needs (NBI) (%)			0.908
0 all basic needs fulfilled	2 (1.8)	1 (0.8)	
1 basic need unfulfilled	18 (16.5)	22 (18.2)	
2 basic needs unfulfilled	52 (47.7)	56 (46.3)	
3 basic needs unfulfilled	32 (29.4)	34 (28.1)	
4 basic needs unfulfilled	5 (4.6)	8 (6.6)	
Households with a smoker (%)	2 (1.7)	5 (3.9)	0.314
Reported frequency of household alcohol consumption (%)			
Household members do not consume alcohol	75 (65.8)	73 (57.5)	0.684
Once a year (at local festivity)	23 (20.2)	30 (23.6)	
Once or less per month	8 (7.0)	13 (10.2)	
2–4 times per month	8 (7.0)	9 (7.1)	
2–3 times per week	-	1 (0.8)	
4 or more times per week	-	1 (0.8)	

Means ± standard deviation for continuous data normally distributed, median [Interquartile range] for continuous data not normally distributed and absolute frequency (percentages) for categorical variables. ICS: Improved cookstove, HDL: High-density lipoprotein, NBI: Unsatisfied basic needs (Spanish abbreviation), masl: metres above sea level. The significance level was set at p ≤0.05.

In respect to the household characteristics of the participants, the proportion of household with electricity was significantly higher in the open fire stove group (82.7%) compared with the ICS group (71.1%). Additionally, 85% of the houses in both groups were built with adobe walls, tile roofs and earthen floors. More than half of the participants in both groups owned a private latrine, and almost all households were equipped with piped water. Additionally, more than 68% of the households had at least one member that participated in a community group or national support programme. The percentage of households in each NBI poverty category was similar in both groups. Overall, the prevalence of smoking was low. In more than half of the households, no one reported to consume alcohol. Excessive alcohol was consumed mainly at local celebrations once a year.

### Dietary characteristics

Overall, 136 different foodstuffs were consumed among all participants. They were categorised in carbohydrates (cereals, grains and tubers), vegetables, fruits, vegetable protein (legumes), animal protein, dairy products and fats. The majority of foods in the list were purchased in the local shops (“*bodegas*”) or at the weekly district market (S2 Appendix). Table 2 presents the counts of each food group and the DDS. Carbohydrates had the highest mean count, followed by vegetables, fat, fruits, and vegetable protein, dairy and animal protein. Dietary diversity using the DDS, had a similar average score in both groups (4.6 in ICS and 4.4. in open fire stove groups). The DDS and the counts per food group did not significantly differ between ICS and open fire stove users.

**Table 2:** Diet Diversity Score (DDS) and frequency of food groups among improved cookstoves and open fire stove users of the provinces of San Marcos and Cajabamba, Cajamarca-Peru.

	ICS users (n = 190)			Open fire stove users (n = 195)			
	Mean ± SD or median (IQR)	Min	Max	Mean ± SD or median (IQR)	Min	Max	P-value
Diet Diversity Score (DDS)	4.6 ± 1.2	1	7	4.4 ± 1.3	1	7	0.131
Counts per food group							
Carbohydrates	6.4 ± 2.0	2	13	6.6 ± 2.1	1	13	0.255
Animal protein	1 (1)	0	3	0 (1)	0	2	0.716
Vegetable protein	1 (1)	0	4	1 (1)	0	3	0.665
Fat	1.5 ± 1.0	0	4	1.5 ± 1.0	0	4	0.481
Fruits	1 (2)	0	4	1 (1)	0	6	0.167
Vegetables	4 (4)	0	15	4 (4)	0	13	0.534
Dairy	0 (1)	0	2	0 (1)	0	3	0.201

ICS: improved cookstove, Min / Max.: minimum and maximum number of Diet Diversity Score and counts per food group in 24-hour food recall, SD: Standard deviation, IQR: interquartile range. The significance level was set at p ≤0.05.

Different carbohydrates, such as beverages with oats, bread, traditional “cachangas” (whole-wheat fried pancakes) and soups with potatoes, pasta, or rice, usually comprised the first meal of the day. Lunch was eaten when family members returned from fieldwork, unless lunch food was taken to the field or provided at the employers’ location. Lunch comprised mostly of rice, beans and potatoes, sometimes served with small portions of vegetables and meat (mostly pig) or eggs. Dinner varied the most among participants. Some families ate leftovers from breakfast or lunch, or just ate bread or “cachangas” with a hot drink (tea or herbal tea). Vegetable oils and lard used for food preparation were the main fats consumed. Fruits were sometimes eaten between meals, but mostly consumed as juices.

### Metabolic syndrome

**Fig 2:**
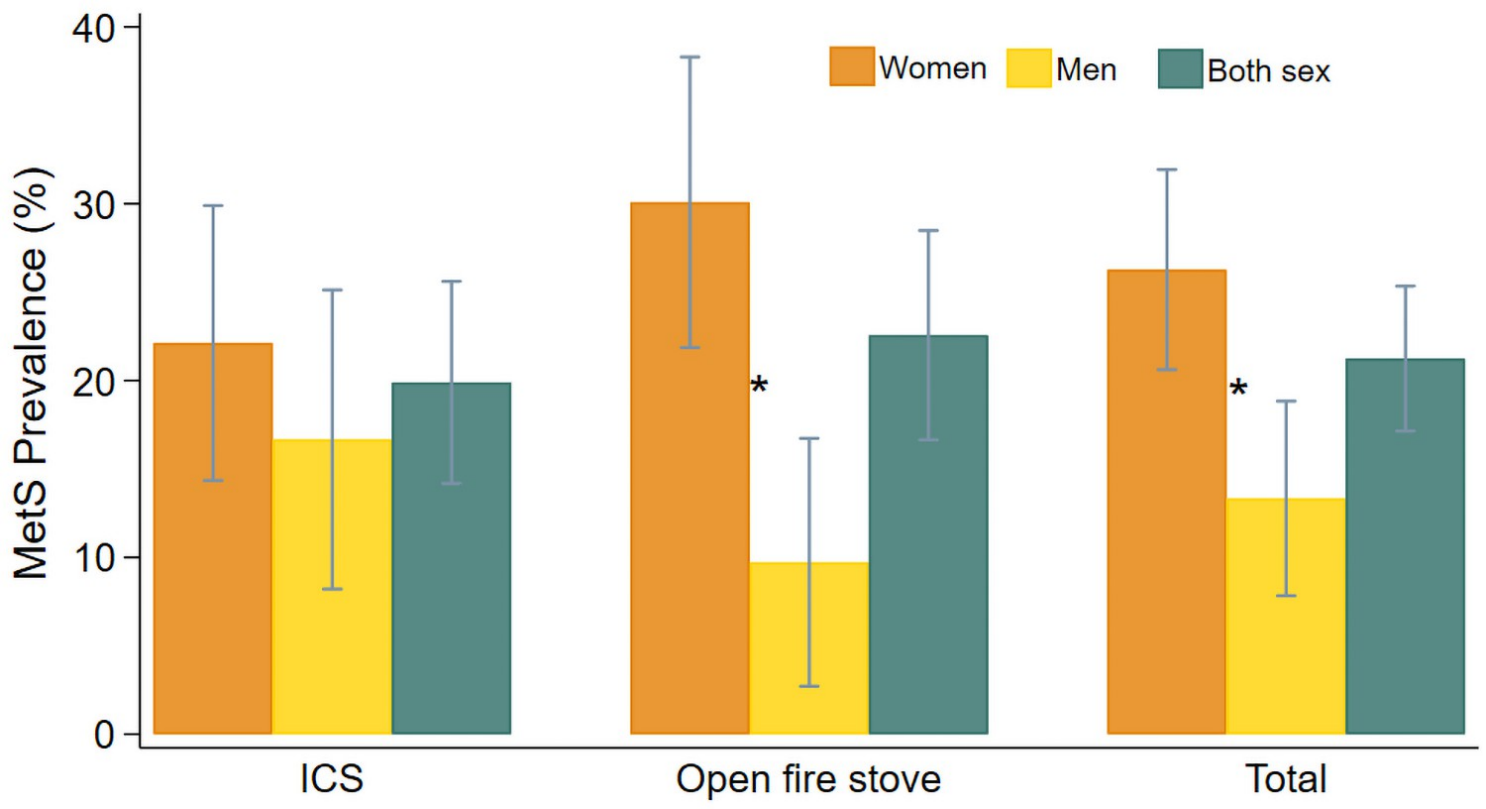
Prevalence and 95% confidence intervals of metabolic syndrome (MetS) among improved coosktove and open fire stove users of the provinces of San Marcos and Cajabamba, Cajamarca-Peru. * p-value < 0.05, Pearson Chi squared test.

Overall, the prevalence of MetS was 21.3% (95% confidence intervals (CI): 17.2–25.4) in all participants, 20.0% (CI: 16.6–28.5) in the ICS group and 22.7% (CI: 14.3–25.7) in the open fire stove users group. Additionally, MetS prevalence was higher in women (26.4%, CI: 20.7–32.1) than in men (13.3%, CI: 7.8–18.8). This trend was also observed within each study group, 22.3% (CI: 14.5–30.2) in women *versus* 16.7% (CI: 8.2–25.1) in men of the ICS group, and 30.1% (CI: 21.9–38.3) in women *versus* 9.7% (CI: 2.7–16.7) in men of the open fire stove group. Pearson Chi squared tests showed that gender differences of MetS prevalence were statistically significant (p = 0.002) in the total population and in the open fire stove group (p = 0.001). In addition, while the prevalence of MetS in women was higher in the open fire stove group compared to the ICS group, we observed the opposite in men (Fig 2).

### Univariable and multivariable models

Table 3 presents the findings from univariable and multivariable Poisson regression models for MetS. The univariable analysis indicated no association between the type of cookstove and the prevalence of MetS (Prevalence Ratio (PR) = 0.89; p = 0.570). Women were almost twice as likely to have MetS compared to men (PR = 1.98; p = 0.002). The PR of MetS significantly increased with age (PR = 1.03; p = 0.021), BMI (PR = 1.20; p<0.001), and decreased for participants living ≥2500 masl (PR = 0.52; p = 0.002).

**Table 3:** Univariable and multivariable analysis of determinants of metabolic syndrome in adults of the provinces of San Marcos and Cajabamba, Cajamarca-Peru.

Determinants	Univariable			Multivariable*		
	PR	(95% CI)	p-value	PR	(95% CI)	p-value
Type of cookstove						
Open fire stove	1.00			1.00		
Improved cookstove (ICS)	0.89	(0.59–1.34)	0.570	0.94	(0.65–1.36)	0.732
Sex						
Men	1.00			1.00		
Women	1.98	(1.30–3.02)	**0.002**	1.36	(0.83–2.23)	0.223
Age (years)	1.03	(1.00–1.05)	**0.021**	1.00	(0.98–1.03)	0.595
Occupation						
Non-agricultural work	1.00					
Agricultural work	0.99	(0.66–1.50)	0.976			
Education level						
Incomplete secondary school	1.00					
Completed secondary school or more	0.89	(0.53–1.50)	0.660			
Altitude of residence						
<2500 masl	1.00					
≥2500 masl	0.52	(0.34–0.79)	**0.002**	0.65	(0.44–0.96)	**0.030**
NBI						
0–2 basic needs fulfilled	1.00					
3–4 basic need fulfilled	0.85	(0.53–1.34)	0.477			
BMI	1.20	(1.17–1.24)	**<0.001**	1.19	(1.14–1.23)	**<0.001**
DDS	1.14	(0.99–1.31)	0.080	1.05	(0.90–1.24)	0.537

DDS: diet diversity score, BMI: body mass index, NBI: Unsatisfied basic needs (Peruvian poverty index), masl: metres above sea level. PR: prevalence ratio, CI: confidence interval. Results with p ≤0.05 are marked in bold.

*This model includes and mutually adjusts for type of cookstove and all variables from the univariable analysis with a p<0.20.

The multivariable analysis including type of cookstove, sex, age, altitude of residence, BMI and DDS did not indicate a statistically significant association between MetS and the type of cookstove, sex, age and DDS. Furthermore, this model exhibited an independent effect of altitude and BMI on MetS: participants living at altitudes ≥2500 masl (PR = 0.65; p = 0.030) had 35% less of a chance of having MetS; and a higher BMI was found among participants with MetS (PR = 1.19; p<0.001).

The multivariable analyses for each individual component comprising MetS are presented in Table 4 and the univariable analysis is found in S3 Appendix. We did not observe an association between any of the five components and the type of cookstove. BMI was the only statistically significantly variable that was associated with higher prevalence of all five MetS components. The prevalence of elevated waist circumference increased slightly with age, and women had a more than four-fold chance of having an elevated waist circumference compared to men. Additionally, living at altitude also decreased participants’ prevalence of elevated glucose levels.

**Table 4:** Multivariable analysis of the determinants of five components of metabolic syndrome in adults of the provinces of San Marcos and Cajabamba, Cajamarca-Peru.

Determinants	Multivariable analysis*														
	Waist circumference			Blood pressure			HDL cholesterol			Triglycerides			Glucose		
	PR	(95% CI)	p-value	PR	(95% CI)	p-value	PR	(95% CI)	p-value	PR	(95% CI)	p-value	PR	(95% CI)	p-value
Type of cookstove															
Open fire stove	1.00			1.00			1.00			1.00			1.00		
ICS	0.95	(0.80–1.12)	0.505	1.25	(0.54–2.90)	0.602	0.96	(0.88–1.04)	0.274	1.17	(0.48–1.70)	0.759	0.81	(0.50–1.33)	0.404
Sex															
Men	1.00			1.00			1.00			1.00			1.00		
Women	3.31	(2.34–4.69)	**<0.001**	0.23	(0.07–0.74)	**0.014**	1.13	(1.01–1.26)	0.030	0.67	(0.41–1.10)	0.114	1.71	(0.93–3.14)	0.086
Age (years)	1.02	(1.01–1.03)	**0.003**	1.03	(0.98–1.08)	0.219	0.99	(0.99–1.00)	**0.023**	0.99	(0.96–1.02)	0.566			
Occupation															
Non-agricultural work															
Agricultural work	0.92	(0.76–1.11)	0.381										0.65	(0.35–1.21)	0.174
Education level															
Incomplete secondary school															
Completed secondary school or more	1.09	(0.84–1.40)	0.522	1.62	(0.58–4.52)	0.356									
Altitude of residence															
<2500 masl															
≥2500 masl	0.87	(0.73–1.03)	0.100										0.39	(0.22–0.70)	**0.001**
NBI															
0–2 basic needs fulfilled				1.00									1.00		
3–4 basic need fulfilled				0.47	(0.16–1.43)	0.185							1.51	(0.94–2.42)	0.086
BMI	1.10	(1.08–1.13)	**<0.001**	1.22	(1.11–1.33)	**<0.001**	1.02	(1.01–1.03)	**0.005**	1.17	(1.13–1.23)	**<0.001**	1.07	(1.01–1.13)	0.027
DDS	1.03	(0.95–1.12)	0.416	**-**			1.03	(0.99–1.06)	0.171	0.92	(0.75–1.12)	0.382			

ICS: improved cookstove, DDS: diet diversity score, BMI: body mass index, NBI: Unsatisfied basic needs (Peruvian Poverty index), PR: prevalence ratio, CI: confidence interval. Results with p ≤0.05 are marked in bold.

*This model includes and mutually adjusts for type of cookstove and all variables from the univariable analysis (S3 Appendix) with a p<0.20.

## Discussion

This study investigated the relationship between metabolic syndrome prevalence and the use of an indoor ventilated improved cookstove in early- and middle-aged adults in the rural Andes. We did not find a statistically significant association between the type of cookstove used and MetS. Moreover, BMI increased the risk of MetS and living at high altitude played an important protective role against MetS in these populations. However, this effect must be taken with caution because the protective impact on health could be from social determinants associated with living at high altitude (i.e. level of urbanisation, access to healthy foods) rather than the physiological response to the high altitude.

Our results indicate no differences in the occurrence of MetS in different stove type users, concurring with findings from a study in Honduras where the prevalence of MetS also did not differ between open fire and ICS users [42]. However, the Honduras study revealed that MetS prevalence increased with air pollution concentrations, regardless of the type of stove used. In both our Peruvian and the Honduran studies, as well as in many other improved biomass cookstove intervention studies, the 24-hour kitchen PM_2.5_ levels in the intervention groups did not reach the World Health Organization air quality standards [28–30, 33, 42–44]. Nonetheless, there is evidence that these interventions do have positive effects on other biomarkers such as blood pressure and inflammatory markers in women over 50 years and pregnant women despite not meeting these standards [28–30]. Contrary to other studies, we found that age was not associated with the prevalence of MetS [21, 24], which might be explained by our early- and middle-aged study population. The latency of the effect of HAP reduction meeting WHO standards on MetS and its impact in different life stages from clean energy cooking is not yet fully understood. Thus, the average follow-up time in this study was 13 months and the HAP reduction may have been too short to establish a beneficial health association between ICS use and MetS in our early- and middle-aged adult population.

Women spend more time in the kitchen than men. However, following the ICS installation resulting in smoke-free and thus, seemingly emission-free kitchen environments, men started to spend more time in the kitchen than before the ICS installation (Authors, 2015 unpublished observation). Thus, we suppose that men increased their exposure to HAP after the ICS installation, which may explain why, even though not significantly, the prevalence of MetS was higher in men of the ICS users group compared the open fire stove users group.

Our analysis showed a higher prevalence of MetS in women compared to men, which is consistent with other studies in other areas of Peru [21–24]. One of these studies suggests that MetS in women could be mainly driven by increased waist circumference, which could be attributed to a sedentary lifestyle [22–24]. There is evidence that the pathophysiology of MetS is sex-specific and that biological characteristics in women (i.e. fat distribution and hormones levels) may contribute to early development of MetS [45]. However, we believe that the difference in men and women MetS prevalence cannot be purely attributed to biological factors, since lifestyle factors such as environmental exposure, physical activity and diet may also differ between sexes.

A sedentary lifestyle is uncommon for Andean population. Both sexes are known to be physically active due to their daily chores and work-intensive subsistence farming. Differences of physical activity between sexes is unknown and future research is necessary to confirm if these populations are meeting the global recommendations for physical activity of 30 minutes of moderate/intensity activity daily [46]. Women in our setting prepare the daily meals, stay at home, carry out household chores and take care of the children and household livestock (including grazing the animals). Women also work attending their fields, as men do, however it could be possible that men do more vigorous and strength demanding activities than women.

We observed that dietary diversity assessed through the DDS appeared not to be a determinant of MetS. However, this score could not measure the quantity of food consumed and the possible sex differences in food intake. Our study showed that BMI is an important risk indicator of metabolic abnormalities and supports the use of BMI assessment in the prevention and control of NCDs in the Peruvian population [21]. Furthermore, there is great need to inform the local population on the risks of high BMI, as they are currently unacquainted with the negative effects of overweight and obesity [21, 47].

This study shows that residing at high altitude was associated with lower prevalence of MetS and elevated glucose levels. Both associations have also been found in Ecuador [48], while a longitudinal study in Peru only confirmed the association between residing at high altitude and low risk of elevated glucose levels [22]. According to the latter, the risk of elevated blood pressure decreased with altitude, but this was not observed in our setting [22, 49]. The high prevalence of MetS in locations at lower altitude could be due to the migration of diagnosed patients with MetS or CVDs or chronic mountain sickness to lower altitude locations as a result of their health condition [50]. However, we do not consider reverse causality as an explanation for our setting because these diseases are usually not routinely assessed during regular health check-ups in these areas due to lack of resources and specialists in remote areas [47]. The majority of the villagers were unacquainted with the concepts, signs and symptoms of MetS or CVDs conditions at large before this study [47].

Altitude has physiological effects on the human body that could influence the development of CVDs. Short term hypoxic exposure slightly increases the variations in heart rate and blood pressure, while long-term hypoxemia decreases blood pressure, and can produce excessive erythrocytosis [50, 51]. Yet, in the analysis of the single components comprising MetS, we did not find an association between altitude and blood pressure, which might indicate that the association of MeS with altitude is not mediated through the blood pressure mechanism.

Also, concurring with others, we speculate that altitude is inversely associated with the level of urbanisation [49]. High levels of urbanisation (usually in lower altitudes) bring about changes in diet, physical activity and socioeconomic status that can negatively impact health [52] and could be reflected in the higher prevalence of MetS and of elevate glucose levels found in lower altitudes in this study. Even though the diet diversity score was not associated with MetS, we observed through our qualitative measures that communities located at higher altitudes had limited access to transportation, and to processed or junk foodstuffs [47]. Additionally, most of high altitude residents work exclusively as farmers, which corresponds to high levels of physical activity through manual labour. In contrast, participants living in lower altitude locations with higher levels of urbanisation had access to more processed foodstuffs and non-physically demanding work opportunities which, might lead to having unhealthy diets and a more sedentary lifestyle.

This study has limitations: 1) the sample size of early- and middle-aged adults was small, 2) we used the stove type as a proxy of HAP, 3) MetS could not be evaluated pre- and post-intervention of ICS, 4) important lifestyle determinants of MetS were not assessed in the analysis, and 5) the potential recall bias of participants answering the 24-food recall. Most studies on MetS and high altitude focus on populations above 45 years of age, where signs and symptoms (and onset) of NCDs disease begin to appear. Since the c-RCT only enrolled parents of young children, the mean age of the study participants (in their mid-30s) was below the age at which MetS usually manifests. Most studies on MetS and high altitude focus on populations above 45 years of age, where signs and symptoms (and onset) of NCDs disease begin to appear. Since the c-RCT only enrolled parents of young children, the mean age of the study participants (in their mid-30s) was below the age at which MetS usually manifests. Additionally, the c-RCT aimed at evaluating children’s health and did not contemplate the measurement of HAP in all study households and the evaluation of parents’ health. However, because this study was embedded within an c-RCT, cofounding due to household characteristics were minimised. Most importantly, the high prevalence of MetS found in this study provides evidence to support the need of NCD studies in these high altitude settings.

In this study, we prioritised time sensitive data collection of blood measurements after fasting and the physical examination. Due to time constraints, we were not able to include a lifestyle questionnaire covering other risk factors related to MetS such as alcohol consumption, physical activity and family medical history at an individual level. The frequency of alcohol consumption found in this study corroborates provincial reports of alcohol intake, but differ from findings in other rural Andean populations [53, 54]. In this setting, alcohol is mainly consumed at local festivities. Being a farmer was used as a proxy for physical activity. Finally, we cannot exclude a potential recall bias because participants may have omitted or added food during the 24-hour recalls. Time constraints did not allow for multiple food recalls as per standard for the CINE’s method [38] to reflect personal intake or direct observation of meal portions to estimate food intake.

For future research, we suggest the assessment of lifestyle determinants (i.e. physical activity, alcohol intake, vegetables and fruit consumption) using standards surveys and quantitative measurement of air pollution environmental and personal exposure. Additionally, the use of biomarkers for dietary assessments such as urinary metabolic profiling could replace recall-dependent diet assessments [55].

## Conclusions

The use of improved cookstoves,—and the reduction of household air pollution expected from it -, had no significant measurable association with MetS among women and men in our high altitude rural Andean setting. Even though that sex was not associated with MetS, we observed differences in the prevalence of MetS in men and women overall and within improved cookstove and open fire stove users groups, which highlights the importance of exploring gendered differences of non-communicable diseases in Andean populations.

Residence at high altitude is also an important protective factor for metabolic syndrome. We speculate that populations residing at lower altitudes closer to urban areas have lower rates of physical activity due to working in more sedentary jobs and have a higher diversity and access to foods high in sugar and saturated fats.

Behavioural differences between women and men and geographic location need to be considered when assessing cardiovascular disease risks in high altitude populations and when developing prevention interventions for these populations.

## Supporting information

S1 Appendix Household air pollution in the kitchen environment of open fire and ICS users. (PDF)

S2 Appendix Lists of foods mentioned in 24-hour food recall. (PDF)

S3 Appendix Univariable analysis of determinants of the five components of metabolic syndrome in in adults of the provinces of San Marcos and Cajabamba, Cajamarca-Peru. (PDF)

S1 Data Study primary data. (XLS)
