# Supplementary material for: Metabolic syndrome in rural Peruvian adults living at high altitudes using different cookstoves
Source: PLoS One. 2022 Feb 8;17(2):e0263415. doi: 10.1371/journal.pone.0263415 (PMC8824363; doi:10.1371/journal.pone.0263415)
Supplement: S1 Appendix — (PDF) [file pone.0263415.s001.pdf]

Metabolic syndrome in rural Peruvian adults living at high altitudes using different cookstoves

Giuliana Sanchez-Samaniego[1,2,3,4], Daniel Mäusezahl[1,2*], Cesar Carcamo[3], Nicole Probst-Hensch[1,2], Héctor Verastegui[3], Stella Maria Hartinger[1,2,3]

1 Department of Epidemiology and Publich Health, Swiss Tropical and Public Health Institute, Swiss TPH, *Basel, Switzerland*

2 University of Basel, *Basel, Switzerland*

3 School of Public Health and Administration, Universidad Peruana Cayetano Heredia, UPCH, *Lima, Peru*

4 Faculty of Science, University of Geneva, *Geneva, Switzerland*

Supporting information 1, Figure: Household air pollution in the kitchen environment of open fire and ICS users

The improved cookstoves (ICS) were installed over six months (Nov. 2015 to Feb. 2016). The community randomised control trial (c-RCT), in which our study was embedded, measured 24-hour $PM_{2.5}$ concentration in a subsample of 40 households (20 open fire stove users and 20 improved cookstove users. The methods used for measuring household air pollution and baseline measurements were published elsewhere (1). Additionally, the c-RCT measured 24-hour $PM_{2.5}$ in the kitchen environment, 4 months (Jun–September, 2016), 8 months (October 2016-February, 2017) and 13 months post installation of the ICS (March-May, 2017).

Figure 1 shows the measurements of 24-h $PM_{2.5}$ concentration in three follow-up measurements after the ICS installation. Consistently, in the first and second follow-ups we observed that median concentration values in the ICS users groups (49.5 $\mu g/m^3$ and 46.4 $\mu g/m^3$, respectively) were almost half of those of the open fire stove group (164.9 $\mu g/m^3$ and 81.0 $\mu g/m^3$, respectively), but the differences were not statistically significant. Furthermore, 24-h $PM_{2.5}$ measurements in the ICS users group did not reach the World Health Organization threshold of 25 $\mu g/m^3$ (2).

Finally, at 13 months post installation the median of $PM_{2.5}$ still differed (78.5 $\mu g/m^3$ in the open fire stove users group versus 100.70 $\mu g/m^3$ in the ICS users group), but the interquartile ranges would still largely overlap. Measurements in both groups include outliers, which may have been caused by the combined used of stoves (stove stacking) or more frequent use of stoves during the cold and rainy season (November to April) than during the dry season from May to October.

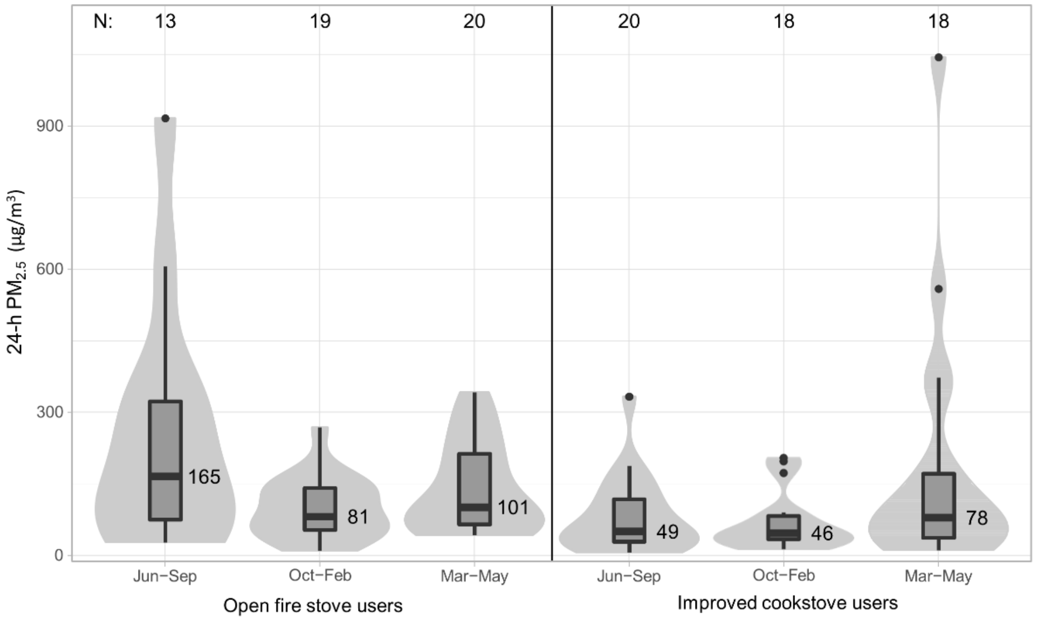

Distribution of 24-hour $PM_{2.5}$ in open fire and improved cookstove users at three follow-up time-points from 2016 to 2017 in the provinces of San Marcos and Cajabamba, Cajamarca-Peru. Violin plots with boxplots and median values.

References

1. Hartinger SM, Nuno N, Hattendorf J, Verastegui H, Karlen W, Ortiz M, et al. A factorial cluster-randomised controlled trial combining home-environmental and early child development interventions to improve child health and development: rationale, trial design and baseline findings. BMC Med Res Methodol. 2020;20(1):73.
2. WHO. WHO Air quality guidelines for particulate matter, ozone, nitrogen dioxide and sulfur dioxide2005.
