# Supplementary material for: Metabolic syndrome in rural Peruvian adults living at high altitudes using different cookstoves
Source: PLoS One. 2022 Feb 8;17(2):e0263415. doi: 10.1371/journal.pone.0263415 (PMC8824363; doi:10.1371/journal.pone.0263415)
Supplement: S2 Appendix — (PDF) [file pone.0263415.s002.pdf]

Metabolic syndrome in rural Peruvian adults living at high altitudes using different cookstoves

Giuliana Sanchez-Samaniego[1,2,3,4], Daniel Mäusezahl[1,2*], Cesar Carcamo[3], Nicole Probst-Hensch[1,2], Héctor Verastegui[3], Stella Maria Hartinger[1,2,3]

1 Department of Epidemiology and Publich Health, Swiss Tropical and Public Health Institute, Swiss TPH, *Basel, Switzerland*

2 University of Basel, *Basel, Switzerland*

3 School of Public Health and Administration, Universidad Peruana Cayetano Heredia, UPCH, *Lima, Peru*

4 Faculty of Science, University of Geneva, *Geneva, Switzerland*

Supporting information 2, Table: List of foods mentioned in the 24-hour food recall

English/Common name	Scientific name	English/Common name	Scientific name
**Cereals and derivates**		**Vegetables**	
Barley (flour, pearl, toasted)	*Hordeum vulgare*	Chard	*Beta vulgaris*
Rice	*Oryza sativa*	Garlic	*Allium sativum*
Pasta		Celery	*Apium graveolens*
Maize (toasted, fresh)	*Zea mays*	Eggplant	*Solanum melongena*
Maize (chochoca)		Broccoli	*Brassica oleracea var.*
Maize flour		Caigua *	*Cyclanthera pedata,*
Purple corn		Scallion	*Allium sp*
Maize mote		Onion	*Allium cepa*
Oat	*Avena sativa*	Cabbage	*Brassica Oleracea*
Quinoa	*Chenopodium quinoa*	Cauliflour	*Brassica oleracea var.*
Wheat (flour, mote, semola, pearl)	*Triticum sp.*	Pumpkin (Chiclayo*)	*Cucurbita ficifolia*
**Fruits**		Spinach	*Spinacia oleracea*
Plum	*Plunus sp.*	Lettuce	*Lactuca sativa*
Fresa	*Strawberry*	Peruvian red pepper	*Capsicum baccatum*
Lemons	*Citrus limon*	Oregan	*Origanu vulgarum*
Lime	*Citrus aurantifolia*	Cucumber	*Cucumis sativus*
Mamey	*Mammea americana*	Pumpkin (zapallo macre**)	*Curcubita maxima*
Mandarine orange	*Citrus reticulata*	Oregan	*Origanu vulgarum*
Mango	*Mangifera sp*	Leek	*Allium ampeloprasum,*
Manzana	*Apple*	Rocoto pepper	*Capsicum pubescens*
Passion Fruit	*Passiflora edulis*	Pumpkin (zapallo macre**)	*Curcubita maxima*
Musk melon	*Cucumis melo*	Amarillo chilli	*Capsicum baccatum*
Quince	*Cydonia oblonga*	Rocoto pepper	*Capsicum pubescens*
Orange	*Citrus aurantium*	Rocoto pepper	*Capsicum pubescens*
Palta	*Avocado*	Pumpkin (zapallo macre**)	*Curcubita maxima*
Papaya	*Carica papaya*	Amarillo chilli	*Capsicum baccatum*
Pear	*Pyrus sp.*	Rocoto pepper	*Capsicum pubescens*
Pineapple	*Ananas comosus*	Tomato	*Solanum lycopersicum*
Peach	*Prunus persica*	Peas	*Pisum sativum*
Banana	*Musa paradisiaca*	Peas	*Pisum sativum*
Grape	*Vitis vinifera*		
**Tubers**		**Legumes and derivates**	
Maca*	*Lepidium meyenii*	Split pea	*Pisum sativum*
Olluco**	*Ullucus tuberosus*	Lentils	*Lens culinaris*
Potatoe	*Solanum tuberosum.*	Bean	*Phaseolus vulgaris*
Chuño* (Freeze-dried potatoe)	*Solanum tuberosum.*	Soybean	*Glycine max*
Cassava	*Manihot esculenta*	Tarwi, chocho*	*Lupinus mutabilis*
Sweet potato	*Ipomoea batatas*	Chickpea	*Cicer arietinum*

Beetroot	*Beta vulgaris*	Faba bean	*Vicia faba*
		Nuña (Bean local variety)	*Phaseolus vulgaris*
English/Common name	***Scientific name***	***English/Common name***	***Scientific name***
**Fish**		**Beverages**	
Canned Tuna		Hierba del Chil *	
Bonito	*Sarda chiliensis*	Lemon verbena	*Aloysia citrodora*
Caballa	*Scomber japonicus*	Hoja d atago*	
Jurel	*Trachurus murphyi)*	Flaxseed	*Linum usitatissimum,*
Lisa	*Mugil cephalus*	Coffee	
**Animal Food**		Tea	
Pig	*Sus domesticus*	Soda	
Rabit	*Allium sativum*	Beer	
Sheep	*Ovis orientalis aries*	Cocoa	
Sheep entrails		Camomile	*Chamaemelum nobile*
Hen	*Gallus bankiva murghi*	Anise	*Pimpinella anisum*
Duck	*Anas. platyrhyncha*	Lemon Balm	*Melissa officinalis*
Chicken	*Gallus bankiva murghi*	Horsetail	*Equisetum sp.*
Chicken liver		Orange-ball-tree	*Buddleja globosa*
Chicken Viscera		Gale of the wind	*Phyllanthus niruri*
Beef	*Bos taurus*	Soda	
Cow entrails		Sugar powder drinks	
Guinea Pig	*Cavia porcellus*	**Spices and herbs**	
Goat kid (Cabrito)	*Capra aegagrus hircus*	Ajinomoto (sodium glutamate)	
Beetroot	*Beta vulgaris*	Cumin	*Cuminum cyminum*
**Dairy products and eggs**		Cinnamon	*Cinnamomum verum*
Milk		Cloves	*Syzygium aromaticum.*
Cheese		Black pepper	*Piper nigrum*
Yogurt		Vinager	
Evaporate milk		Turmeric	*Curcuma longa*
Eggs		Malva	*Malva sp.*
**Oils**		Chiche/wacatay*	*Tagetes minuta*
Cacao		Basil	*Ocimum basilicum*
Peanut		Coriander	*Coriandrum sativum*
Lard		Muña*	*Minthostachys mollis*
Vegetable oils		Wormseed	*Dysphania ambrosioides*
**Others**		Parsley	*Petroselinum crispum*
Mermelade		Peppermint	*Mentha piperita*
Jelly		Ginger	*Zingiber officinale*
Cracker			
Cakes			
Sugar			

*** local name**
