# Supplementary material for: Metabolic syndrome in rural Peruvian adults living at high altitudes using different cookstoves
Source: PLoS One. 2022 Feb 8;17(2):e0263415. doi: 10.1371/journal.pone.0263415 (PMC8824363; doi:10.1371/journal.pone.0263415)
Supplement: S3 Appendix — (PDF) [file pone.0263415.s003.pdf]

Metabolic syndrome in rural Peruvian adults living at high altitudes using different cookstoves

Giuliana Sanchez-Samaniego[1,2,3,4], Daniel Mäusezahl[1,2*], Cesar Carcamo[3], Nicole Probst-Hensch[1,2], Héctor Verastegui[3], Stella Maria Hartinger[1,2,3]

1 Department of Epidemiology and Publich Health, Swiss Tropical and Public Health Institute, Swiss TPH, *Basel, Switzerland*

2 University of Basel, *Basel, Switzerland*

3 School of Public Health and Administration, Universidad Peruana Cayetano Heredia, UPCH, *Lima, Peru*

4 Faculty of Science, University of Geneva, *Geneva, Switzerland*

Supporting information 3, Table: Univariable analysis of determinants of the five components of metabolic syndrome in in adults of the provinces of San Marcos and Cajabamba, Cajamarca-Peru.

Metabolic syndrome (MetS) is a cluster of five risk factors (elevated triglycerides, reduced HDL cholesterol, elevated blood pressure, elevated glucose levels and elevated waist circumference) that increase the risk of developing cardiovascular diseases and type II diabetes. Table 1 presents the mixed effect Poisson regressions for the univariable analysis of determinants of the five components of MetS. We selected the participant's household and place of the physical examination as random effects. As fixed effects we included type of cookstove (Open fire stove versus Improved Cookstove Stove (ICS)), sex, age, agricultural work as main activity, education level, altitude of residence, Unsatisfied Basic Needs (NBI, Spanish abbreviation), body mass index (BMI) and Diet Diversity Score (DDS) in the model.

We found that four main independent variables were associated with three components of MetS. Women had a higher prevalence ratio of elevated waist circumference, low HDL cholesterol, elevated glucose levels, and a lower prevalence ratio of elevated blood pressure compared to men. Agricultural work and living ≥2500 masl (metres above sea level) were protective factors for elevated glucose. Participants living > 2500 masl were also less likely to have elevated waist circumference. Age slightly increased the prevalence for elevated waist circumference and blood pressure. Finally a high diet diversity score (DDS) was associated with a higher prevalence of elevated waist circumference and triglycerides.

S3 Table: Univariable analysis of determinants of the five components of metabolic syndrome in in adults of the provinces of San Marcos and Cajabamba, Cajamarca-Peru.

Determinants	WC			HP			HDL			TRIG			GLUC		
	PR	(95% CI)	p-value	PR	(95% CI)	p-value	PR	(95% CI)	p-value	PR	(95% CI)	p-value	PR	(95% CI)	p-value
Type of cookstove															
Open fire stove	1.00			1.00			1.00			1.00			1.00		
ICS	0.87	(0.72-1.05)	0.157	1.27	(0.50-3.20)	0.614	0.94	(0.87-1.02)	0.109	1.03	(0.66-1.60)	0.908	0.80	(0.47-1.34)	0.385
Sex															
Men	1.00			1.00			1.00			1.00			1.00		
Women	3.74	(2.63-5.31)	**<0.001**	0.30	(0.12-0.76)	**0.011**	1.22	(1.10-1.35)	**<0.001**	0.89	(0.60-1.33)	0.577	1.87	(1.108-3.24)	**0.025**
Age (years)	1.01	(1.00-1.03)	**0.027**	1.08	(1.03-1.13)	**0.001**	0.99	(0.99-1.00)	**0.007**	1.02	(0.99-1.05)	0.141	1.00	(0.97-1.04)	0.794
Occupation															
Non agricultural work	1.00			1.00			1.00			1.00			1.00		
Agricultural work	0.83	(0.66-1.05)	0.115	0.99	(0.40-2.47)	0.987	1.03	(0.95-1.12)	0.484	1.29	(0.85-1.97)	0.231	0.53	(0.29-0.98)	**0.041**
Education level															
Incomplete secondary school	1.00			1.00			1.00			1.00			1.00		
Completed secondary school or more	0.77	(0.57-1.04)	0.0085	1.97	(0.76-5.07)	0.160	0.97	(0.87-1.08)	0.536	1.05	(0.62-1.76)	0.866	0.59	(0.27-1.28)	0.184
Altitude of residence															
<2500 masl	1.00			1.00			1.00			1.00			1.00		
≥2500 masl	0.74	(0.62-0.89)	**0.002**	0.65	(0.25-1.74)	0.393	1.00	(0.93-1.08)	0.982	0.75	(0.48-1.17)	0.208	0.34	(0.19-0.60)	**<0.001**
NBI															
0-2 basic needs fulfilled	1.00			1.00			1.00			1.00			1.00		
3-4 basic need fulfilled	0.94	(0.76-1.16)	0.549	0.42	(0.12-1.40)	0.156	1.01	(0.92-1.09)	0.924	0.75	(0.45-1.24)	0.264	1.49	(0.90-2.46)	0.119
BMI	1.14	(1.11-1.17)	**<0.001**	1.15	(1.07-1.23)	**<0.001**	1.02	(1.01-1.03)	**0.002**	1.15	(1.11-1.19)	**<0.001**	1.10	(1.04-1.16)	**0.001**
DDS	1.19	(1.10-1.28)	**<0.001**	0.98	(0.66-1.46)	0.913	1.04	(1.01-1.08)	**0.021**	0.92	(0.77-1.10)	0.382	1.13	(0.93-1.38)	0.216

WC: elevated waist circumference, HDL: low HDL cholesterol, TRIG: elevated triglycerides levels, GLUC: elevated glucose levels, BP: elevated blood pressure, DDS: dietary diversity score, BMI: body mass index, ICS: improved cookstove intervention. masl: metres above sea level, NBI: Unsatisfied basic needs (Spanish abbreviation), CI: confidence interval. Results with p ≤0.05 are marked in bold.
